# miR-125b-5p/STAT3 Pathway Regulated by mTORC1 Plays a Critical Role in Promoting Cell Proliferation and Tumor Growth

**DOI:** 10.7150/jca.33696

**Published:** 2020-01-01

**Authors:** Chengcheng Zhang, Xiaofeng Wan, Sisi Tang, Kun Li, Yani Wang, Yujie Liu, Quan Sha, Xiaojun Zha, Yehai Liu

**Affiliations:** 1 Department of Otorhinolaryngology, Head & Neck Surgery, The First Affiliated Hospital of Anhui Medical University, Hefei, China; 2 Department of Biochemistry & Molecular Biology, School of Basic Medicine, Anhui Medical University, Hefei, China; 3 The First Clinical Medical College, Anhui Medical University, Hefei, China; 4 Department of Immunology & Allergy and Immunology Research Center, School of Basic Medicine, Anhui Medical University, Hefei, China

**Keywords:** mTOR, miR-125b-5p, STAT3, tumorigenesis

## Abstract

Aberrant activation of the mammalian target of rapamycin complex 1 (mTORC1) plays a critical role in tumorigenesis. However, the precise underlying mechanism is still not fully understood. Although accumulating evidence suggests that mTORC1 signaling is regulated by microRNAs (miRNAs), whether miRNAs are involved in the tumorigenesis mediated by mTORC1 dysregulation remains largely unclear. In our study, the comparison between tuberous sclerosis complex 1 (Tsc1) -/- or Tsc2-/- mouse embryonic fibroblasts (MEFs) and the control cells revealed the involvement of microRNA-125b-5p (miR-125b-5p) in the tumorigenesis driven by mTORC1 activation. Our study also showed that loss of TSC1 or TSC2 led to significant downregulation of miR-125b-5p and upregulation of signal transducer and activator of transcription 3 (STAT3) via mTORC1 activation. Overexpression of miR-125b-5p inhibited the proliferation of the cells with hyperactivated mTORC1 both *in vitro* and *in vivo*. Furthermore, we demonstrated that STAT3 is a direct target of miR-125b-5p. Depletion of STAT3 mimicked the effect of ectopic expression of miR-125b-5p, and reintroduction of STAT3 rescued the compromised cell proliferation driven by miR-125b-5p overexpression in Tsc1-/- or Tsc2-/- MEFs. We conclude that the miR-125b-5p/STAT3 pathway plays a crucial role in hyperactivated mTORC1-mediated tumorigenesis and miR-125b-5p is a potential therapeutic target.

## Introduction

Mammalian target of rapamycin (mTOR), a highly conserved serine/threonine protein kinase, is the master effector in the receptor tyrosine kinase (RTK)/phosphatidylinositol 3-kinase (PI3K)/AKR mouse T-cell lymphoma oncoprotein (AKT)/mTOR signaling pathway via integrating multiple inputs, such as growth factor and nutrient status to orchestrate a number of cellular processes, including cell growth, motility, and survival 1, 2. mTOR can associate with different binding partners to form two functional complexes, mTOR complex 1 (mTORC1) and mTOR complex 2 (mTORC2) 1, 3. mTORC1 is sensitive to rapamycin and activated by RTK/PI3K/ AKT pathway, whereas mTORC2 is rapamycin-insensitive 1. Due to gain-of-function mutations of proto-oncogenes such as epidermal growth factor receptor (EGFR), PI3K, and AKT, or loss-of-function mutations of tumor suppressors, such as phosphatase and tensin homolog deleted from chromosome 10 (PTEN), tuberous sclerosis complex 1 (TSC1), and tuberous sclerosis complex 2 (TSC2), mTORC1 is frequently activated in human cancers 4, 5. TSC2, as a GTPase-activating (GAP) protein, binds with TSC1 to form a complex which negatively regulates a small GTPase, Ras homologue enriched in brain (Rheb) 6. Disruption of TSC1/TSC2 complex by the activated PI3K/AKT pathway, or by inactivating mutations in either *TSC1* or *TSC2*, leads to the accumulation of GTP-bound Rheb, which in turn activates mTORC1 6. Hyperactivated mTORC1 signaling leads to uncontrolled cell growth and tumorigenesis, but the precise mechanisms remain to be further elucidated.

miRNAs are small non-coding, cellular RNAs (21-25 nucleotides) that function as negative regulators of protein-coding genes in multiple cellular processes such as cell proliferation, differentiation and survival 7. In order to regulate expression of their target mRNAs, miRNAs must be assembled into a complex termed the RNA induced silencing complex (RISC) 8. Once assembled, they inhibit the post-transcriptional translation or enhance the cleavage of their target mRNAs through base paring with their 3' untranslated region (3'-UTR) 8. With respect to carcinogenesis, miRNAs can act as either oncogenes or tumor suppressors, depending on the genes they regulate and the cellular context 9. Numerous studies have shown that dysregulation of miRNAs plays an essential role in tumorigenesis through modulation of AKT/mTORC1 signaling pathway^10^. However, the function of miRNAs in mTORC1-induced tumorigenesis remains largely unknown.

In the present study, we demonstrated that activation of mTORC1 downregulated the expression of microRNA-125b-5p (miR-125b-5p). Moreover, miR-125b-5p displayed a tumor suppressive role in the cells with hyperactivated mTORC1 via targeting signal transducer and activator of transcription 3 (STAT3)*.* We suggest that the miR-125b-5p/STAT3 pathway can serve as a potential target for the treatment of cancers associated with dysregulated mTORC1.

## Materials and Methods

### Reagents, plasmids, and antibodies

Rapamycin was obtained from Selleck Chemicals (Houston, TX, USA). Lipofectamine 2000 and NuPAGE 4-12 % Bis-Tris gel were purchased from Life Technologies (Carlsbad, CA, USA). pLXIN-myristoylated AKT1 (myrAKT1) vector and the empty vector pLXIN have been described previously 4. pBabe-puro and pBabe-puro-STAT3 vectors were kindly provided by Dr. Yu Zhang (Cancer Institute & Hospital, Chinese Academy of Medical Sciences). GP-miRGLO firefly luciferase vector was from GenePharma (Shanghai, China). Antibodies specific to p-S6 (Ser235/236) (#4857), S6 (#2317), TSC1 (#6935), Raptor (#2280), Rictor (#2114), p-STAT3 (Tyr705) (#9131), STAT3 (#9139), p-AKT (Ser473) (#4051), and GAPDH (#2218) were obtained from Cell Signaling Technology (Danvers, MA, USA). TSC2 (#4308) and β-actin (#4967) antibodies were obtained from Santa Cruz Technology (Santa Cruz, CA, USA).

### Cell culture and drug treatment

All the mouse embryonic fibroblasts (MEFs), including Tsc1+/+, Tsc1-/-, Tsc2+/+, Tsc2-/-, Pten+/+, Pten-/- and pLXIN or pLXIN-hTSC2 retroviruses infected Tsc2-/- MEFs, have been described previously 4. The retroviral packaging cell line PT67 were purchased from Clontech (Mountain View, CA, USA). HEK 293T cells were obtained from the ATCC (Manassas, VA, USA). All cells were cultured in DMEM supplemented with 10% fetal bovine serum at 37 °C in a humidified incubator containing 5% CO_2_. The DMSO (Sigma, St. Louis, MO, USA) stock of rapamycin was diluted in cell culture medium to a working concentration of 50 nM. Prior to treatment, cells were plated in 12-well plates at a density of 2×10^5^ cells/well and cultured overnight. After 24 h incubation of rapamycin, cells were harvested for quantitative RT-PCR or western blot analyses as described below.

### Retroviral and lentiviral transduction

Production of retroviruses and subsequent generation of stable gene expression cell lines have been described previously 4. In brief, pLXIN-myrAKT1, pLXIN, pBabe-puro-STAT3 or pBabe-puro vectors were transfected into the retroviral packaging cell line PT67 using Lipofectamine 2000. After filtered through a 0.45 μm filter (Millipore, Billerica, MA, USA), the conditional culture medium containing viruses were used to infect target cells. The transduced cells were selected with 2-5 μg/mL puromycin for stably expressing cells.

The lentiviral vector GV369 expressing miR-125b-5p and the empty vector were obtained from GeneChem (Shanghai, China) and were termed LV-miR-125b-5p and LV, respectively. The GV248 lentiviral shRNA expression vector targeting mouse Raptor, mouse Rictor, and the control scrambled shRNA (shSc) were obtained from GeneChem (Shanghai. China). The target sequences were as follows: shRaptor, 5'-GGACAACGGTCACAAGTAC-3'; shRictor, 5'-GCCCTACAGCCTTCATTTA-3'; shSTAT3, 5'-CTGGATAACTTCATTAGCA-3'; shSc, 5'-AATCGCATAGCGTATGCCG-3'. Lentiviruses were generated by transfecting with either of the recombinant vectors or the corresponding control vectors together with packaging plasmids (pVSVG, pREV, and pMDL) into HEK 293T cells. Culture supernatants were collected after 48-72 h of transfection and then filtered through a 0.45 μm filter for infection of target cells as described previously 11. In brief, cells were seeded into 6-well plates and transfected with lentivirus with a multiplicity of infection (MOI) of 10-20. 12 h after infection, the medium was replaced with fresh complete growth medium. Cells were continued to grow for 72 h and then collected for quantitative real-time PCR and western blot analyses.

### Western blot

Total cellular proteins were extracted by RIPA buffer (Beyotime Biotechnology, Haimen, China). Immunoblotting analysis was performed as described previously 11. In brief, whole cell or tissue lysates were separated by NuPAGE 4-12 % Bis-Tris gels (Life Technologies), transferred to PVDF membrane (Millipore), and then incubated with the primary and secondary antibodies. The bands were visualized using Pierce^TM^ ECL Western Blotting Substrate (Thermo Fisher Scientific, Waltham, MA, USA) according to the manufacturer's instructions.

### Quantitative real-time PCR (qRT-PCR)

Total RNA from cells and tissues were isolated using TRIzol reagent (Life Technologies) according to the manufacturer's instructions. For mRNA quantification, first-strand cNDA synthesis was performed using the RevertAid™ First Stand cDNA Synthesis Kit (Fermentas, Waltham, MA, USA) according to the protocol provided by the manufacturer. qRT-PCR detection of transcripts was performed using SYBR Premix Ex Taq^TM^ II (TaKaRa, Shiga, Japan). The expression of miR-125b-5p was detected using the Hairpin-it miRNAs qPCR Quantitation Kit (GenePharma) according to the producer's instructions. qRT-PCR was performed on the ABI Prism 7500 fast Sequence Detection System (Applied Biosystem, Foster City, CA, USA). β-actin or U6 served as an internal control. The primers used were as follows: STAT3 forward: 5'-AGCTGGACACACGCTACCT-3'; and reverse: 5'-AGGAATCGGCTATATTGCTGGT-3'. β-actin forward: 5'-AGAGGGAAATCGTGCGTGAC-3'; and reverse: 5'-CAATAGTGATGACCTGGCCGT-3'. miR-125b-5p forward: 5'-ACTGATAAATCCCTGAGACCCTAAC-3'; and reverse: 5'-TATGGTTTTGACGACTGTGTGAT-3'. U6 forward: 5'-CAGCACATATACTAAAATTGGAACG-3'; and reverse: 5'-ACGAATTTGCGTGTCATCC-3'.

### miRNA transfection

All the RNA oligonucleotides were synthesized by GenePharma (Shanghai, China). The sequences are listed as follows: miR-125b-5p mimics, 5'-UCCCUGAGACCCUAACUUGUCA-3'; mimics negative control, 5'-UUCUCCGACGUGUCACGUTT-3'; miR-125b-5p inhibitor, 5'-UCACAAGUUAGGGUCUCAGGGA-3'; and inhibitor negative control, 5'-CAGUACUUUUGUGUAGUACAA-3'. Cells were seeded in a 12-well plate, incubated overnight, and then transfected with miR-125b-5p mimics (50 nM), miR-125b-5p inhibitor (200 nM), or their corresponding controls using Lipofectamine 2000 according to the protocol provided by the manufacturer. Cells were harvested 48 h after transfection for further analysis by qRT-PCR or western blot as described above.

### miRNA expression profiling

Total RNA was isolated using the mirVana miRNA isolation kit (Ambion, Austin, TX, USA) according to the manufacturer's instructions. miRNA microarray analysis was performed using the miRCURY LNA Array (version 11.0 Exiqon) at KangChen Bio-Tech Corporation (Shanghai, China). miRNAs were considered to be differentially expressed if the difference in its expression was more than 2.0-fold.

### Reporter constructs and Luciferase reporter assay

A fragment of 61 bp STAT3 3´-untranslated region (3´-UTR) containing the putative binding site for miR-125b-5p was generated by annealing oligonucleotides 5'-CCTGCCTCAGACTACAGGCCCTCAGCAAAGCTCAGGGAGTATGGTCCTTATTCTATGCGCTTC-3' and 5'-TCGAGAAGCGCATAGAATAAGGACCATACTCCCTGAGCTTTGCTGAGGGCCTGTAGTCTGAGGCAGGAGCT-3', cloned into the *Sac*I and *Xho*I sites of GP-miRGLO firefly luciferase vector (GenePharma), called STAT3-WT. Similarly, the following primers: 5'-CCTGCCTCAGACTACAGGCCCTCAGCAAAGGAGTCCCAGTATGGTCCTTATTCTATGCGCTTC-3' and 5'-TCGAGAAGCGCATAGAATAAGGACCATACTGGGACTCCTTTGCTGAGGGCCTGTAGTCTGAGGCAGGAGCT-3' were annealed and inserted into the GP-miRGLO vector, which generated a mutation of 7 bps from CTCAGGG to GAGTCCC in the predicted miR-125b-5p target binding site, called STAT3-Mut. All constructs were confirmed by DNA sequencing. For luciferase reporter assay, Tsc2-/- MEFs were plated in a 24-well plate at about 70% confluence. Then, cells were transfected with miR-125b-5p mimics or mimics negative control (NC), together with luciferase reporter plasmids (STAT3-WT or STAT3-Mut) using Lipofectamine 2000. 48 h after transfection, luciferase activities were measured using the Dual-Luciferase Reporter assay system (Promega, Madison, WI, USA), according to the manufacturer's procedures. Renilla luciferase activity was used as a control. Each sample was assayed in triplicate.

### Cell proliferation assay

Cell proliferation was measured by Cell Counting Kit-8 (CCK-8) (Beyotime Biotechnology) as described previously 11. In brief, cells (2.0×10^3^ per well) were seeded into 96-well plates in triplicate and the proliferation was monitored for up to 4 days. The absorbance at 570 nm was evaluated using a spectrophotometer (Thermo Fisher Scientific).

### Colony formation assay

Cells were seeded into 10 cm cell culture dish at a density of 300 cells per dish. The cells were cultured for about 2-3 weeks in DMEM medium containing 10 % FBS. After washed in PBS, the cells were fixed in 4% formaldehyde for 15 min and stained with 0.1 % crystal violet (Sigma) for 15 min. Pictures were captured using an optic microscope (Olympus, Tokyo, Japan). The number of colonies with over 50 cells was counted.

### Mouse kidney tumor assessment

The kidney cystadenoma tissues and the adjacent normal tissues from three mice with heterozygous deletion of Tsc2 (Tsc2+/-, C57BL/6, 17-18 months old) were sonicated and extracted for immunoblotting or qRT-PCR analyses as described above.

### Tumorigenicity in nude mice

Five-week old immunodeficient BALB/c nude mice (male) were purchased from Vital River Laboratory Animal Technology (Beijing, China). For tumorigenicity assays of the cells with miR-125b-5p overexpression, 4×10^6^ LV-miR-125b-5p or LV lentiviruses transduced Tsc1-/- MEFs in 0.2 ml DMEM were inoculated subcutaneously into the right anterior armpit of mice and the tumor growth was monitored. Six mice were used in each cohort. For tumorigenicity assays of the cells with STAT3 depletion, 4×10^6^ Tsc2-/- MEFs transduced with shSTAT3 or shSc lentiviruses in 0.2 ml DMEM were inoculated subcutaneously into the flanks of mice (n=3) as the indicated. Tumor growth was examined every 5 days, and tumor volume was calculated using the equation: volume (mm^3^) = (length×width^2^)/2. The animals were sacrificed by CO_2_ euthanasia on day 60 after inoculation. After taking photos, the tumor tissues were harvested, weighted, and then for further analysis. All animals were maintained and used in strict accordance with the guidelines of the Animal Center of Anhui Medical University, and all animal experimental procedures were approved by the Experimental Animal Ethical Committee of Anhui Medical University.

### Immunohistochemistry (IHC) analysis

Tumor tissues were fixed in 4% paraformaldehyde, embedded in paraffin, and then cut into 4 μm slides. The histological sections were stained with hematoxylin-eosin (H&E) or immunostained with rabbit monoclonal antibodies against Ki-67 (Abcam, Cambridge, MA, USA) according to the manufacturer's protocols. All sections were photographed at a magnification of ×200.

### Statistical analysis

Data were analyzed using Student's t test (2-tailed) or One-way ANOVA as appropriate with GraphPad Prism 5 software (GraphPad Software Inc., San Diego, CA, USA). Statistical significance was defined as *P* < 0.05.

## Results

### Loss of TSC1 or TSC2 upregulates the expression of STAT3

**Figure 1:**
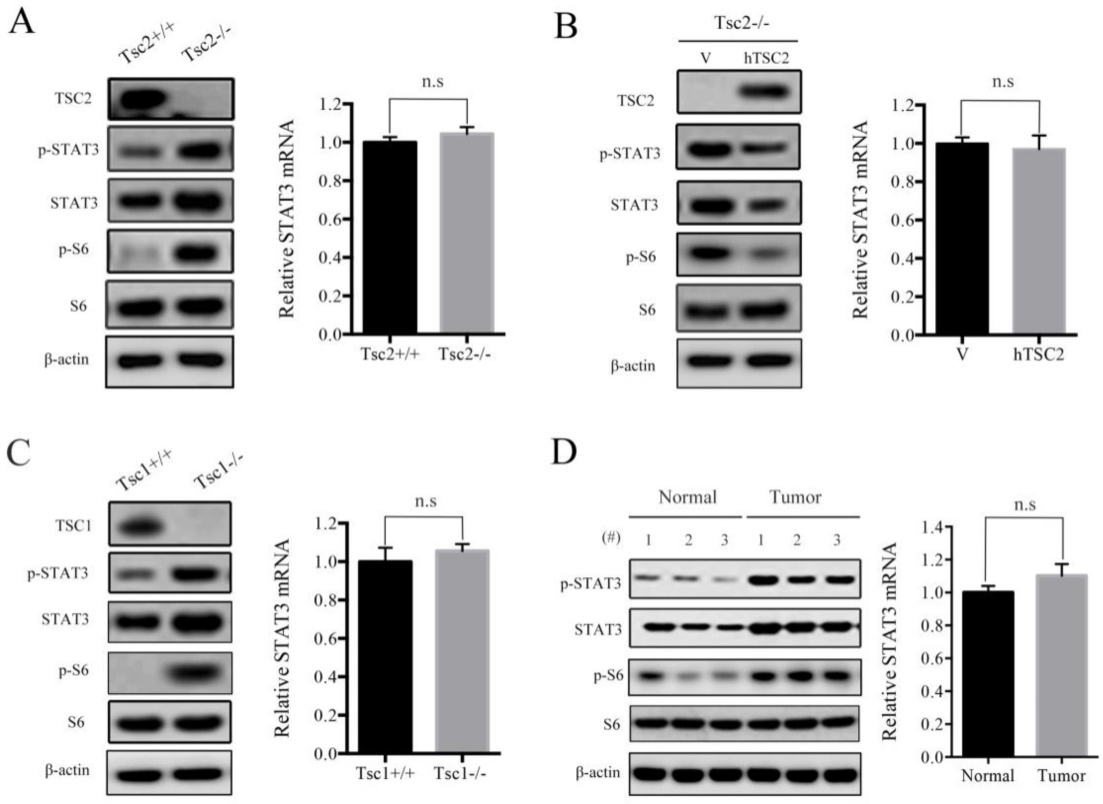
** Loss of TSC1 or TSC2 upregulates the expression of STAT3.** (A) Tsc2+/+ and Tsc2-/- MEFs. (B) Tsc2-/- MEFs were transduced with the retroviruses for human TSC2 (hTSC2) in pLXIN or its control vector pLXIN (V). (C) Tsc1+/+ and Tsc1-/- MEFs. (D) Kidney cystadenomas (Tumor) and the adjacent normal tissues (Normal) from Tsc2+/- mice (n=3). (A-D) Cell lysates were subjected to immunoblotting with the indicated antibodies (left panels). STAT3 mRNA was analyzed by qRT-PCR (right panels). n.s indicates no significant difference.

It has been shown that STAT3 was aberrantly activated or overexpressed in many types of cancers 12. We have previously reported that hyperactivated mTORC1, due to loss of either TSC1 or TSC2, led to activation of STAT3 4, 13, 14. However, the exact mechanisms underlying mTORC1 regulation of STAT3 remains largely obscure. Herein, we first checked the protein and mRNA levels of STAT3 in Tsc2-/- MEFs and the control cells (Tsc2+/+ MEFs). As expected, western blot analysis demonstrated that loss of TSC2 led to activation of mTORC1 signaling (p-S6 is a marker of mTORC1 activity) and upregulation of phospho-STAT3 Tyr^705^ (p-STAT3) (Figure 1A left panel). Interestingly, the STAT3 protein level but not its mRNA level was significantly upregulated in Tsc2-/- MEFs compared with Tsc2+/+ MEFs (Figure 1A), indicating that loss of TSC2 enhanced STAT3 expression not at the transcriptional level. Furthermore, ectopic expression of wild-type human TSC2 (hTSC2) normalized the protein level of STAT3 (Figure 1B left panel), but had no effect on the mRNA level of STAT3 in Tsc2-/- MEFs (Figure 1B right panel). Consistent with those observations, p-STAT3 and STAT3 protein, but not STAT3 mRNA level, were substantially increased in Tsc1-/- MEFs as compared to Tsc1+/+ MEFs (Figure 1C). The relationship between TSC1/TSC2 complex and STAT3 was further confirmed *in vivo* by assessing the protein and mRNA levels of STAT3 in renal tumors and adjacent normal renal tissues from Tsc2+/- mice. As shown in Figure 1D, STAT3 protein level, but not its mRNA level was increased within the tumors compared with the adjacent normal tissues. Taken together, loss of TSC1/TSC2 complex elevates the expression of STAT3 at the posttranscriptional level.

### Loss of TSC1/TSC2 complex led to downregulation of miR-125b-5p expression

**Figure 2:**
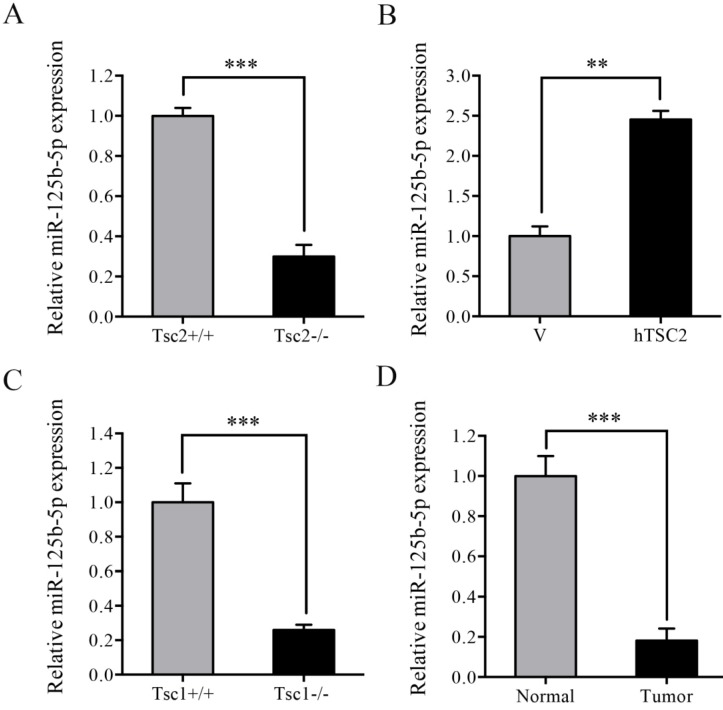
** Loss of TSC1 or TSC2 reduces the expression of miR-125b-5p.** (A) Tsc2+/+ and Tsc2-/- MEFs. (B) pLXIN (V) or pLXIN-hTSC2 retroviruses infected Tsc2-/- MEFs. (C) Tsc1+/+ and Tsc1-/- MEFs. (D) Kidney cystadenomas (Tumor) and the adjacent normal tissues (Normal) from Tsc2+/- mice (n=3). The expression of miR-125b-5p was detected by qRT-PCR. Error bars indicate mean ± SD of triplicate samples. **P<0.01; ***P<0.001.

Since that STAT3 was post-transcriptionally upregulated by loss of TSC1 or TSC2 and modulation by miRNAs has emerged as a critical post-transcriptional mechanism regulating gene expression, we hypothesized that miRNAs was involved in the upregulation of STAT3 in Tsc2-/- or Tsc1-/- MEFs. Subsequently, genome-wide miRNA expression analysis was performed to analyze the difference of the miRNA expression profiles between Tsc2-/- and Tsc2+/+ MEFs. The result showed that there were 13 miRNAs with upregulated expression and 38 miRNAs with downregulated expression in Tsc2-/- MEFs as compared to Tsc2+/+ MEFs (fold change >2) (Table 1). Among the differentially expressed miRNAs, the expression level of miR-125b-5p was significantly decreased in Tsc2-null MEFs and this finding was confirmed by qRT-PCR analysis (Figure 2A). Moreover, reintroduction of hTSC2 robustly restored the expression of miR-125b-5p in Tsc2-/- MEFs (Figure 2B). Loss of TSC1 also led to markedly decreased expression of miR-125b-5p (Figure 2C). In addition, miR-125b-5p expression was reduced in renal tumor tissues as compared to the adjacent normal renal tissues from Tsc2+/- mice (Figure 2D). Collectively, TSC1/TSC2 complex positively regulates miR-125b-5p expression.

### mTORC1 upregulates STAT3 and downregulates miR-125b-5p expression

**Figure 3:**
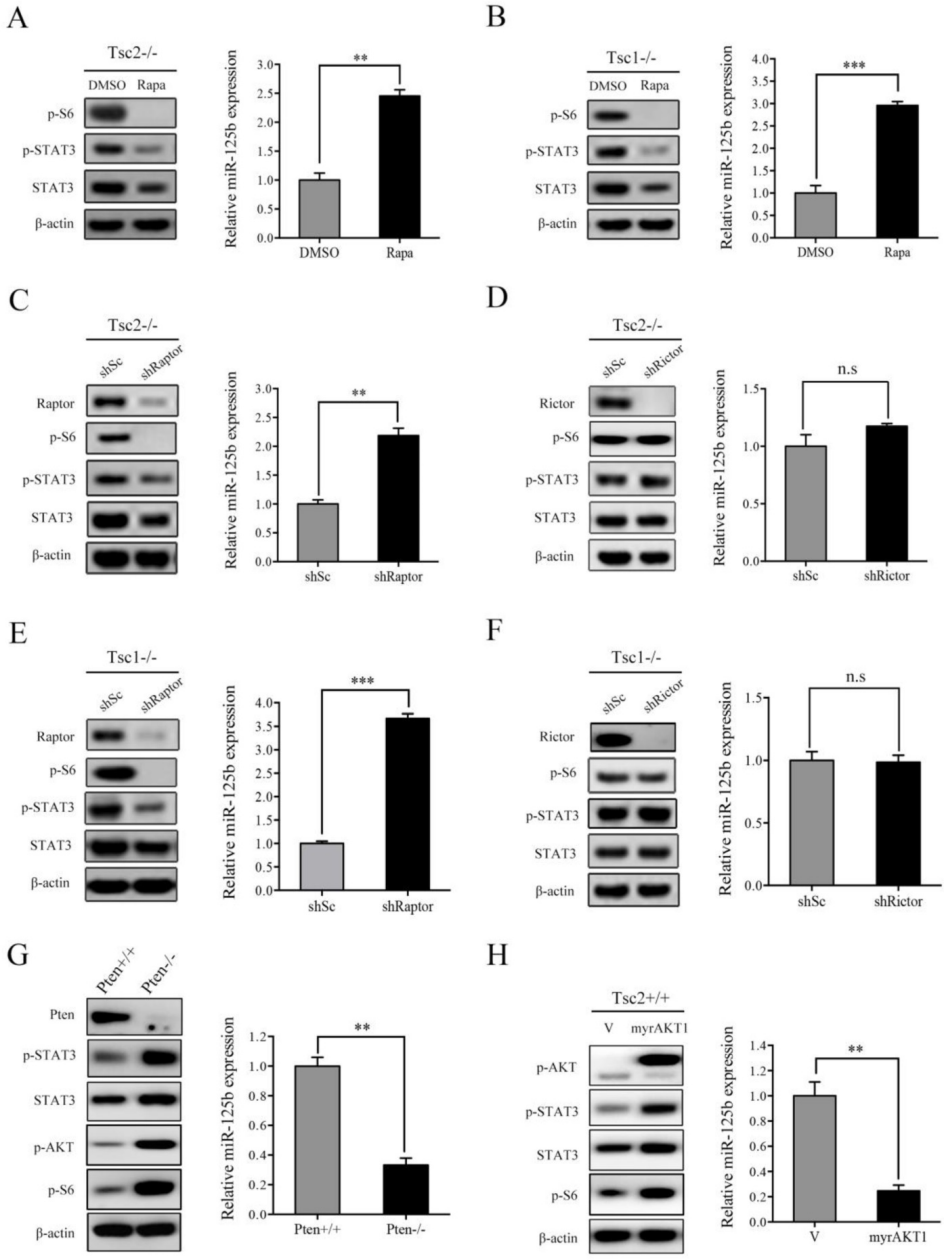
** mTORC1 upregulates STAT3 and downregulates miR-125b-5p expression.** (A, B) Tsc2-/- MEFs (A) or Tsc1-/- MEFs (B) were treated with DMSO or with 50 nM rapamycin (Rapa) for 24 h. (C, E) Tsc2-/- MEFs (C) or Tsc1-/- MEFs (E) were infected with lentiviruses harboring vectors encoding Raptor shRNA (shRaptor) or the control shRNA (shSc). (D, F) The shRNA that silences Rictor (shRictor) or a control shRNA (shSc) were stably expressed in Tsc2-/- MEFs (D) or Tsc1-/- MEFs (F). (G) Pten+/+ and Pten-/- MEFs. (H**)** Tsc2+/+ MEFs were transduced with the retroviruses for myrAKT1 in pLXIN or its control vector pLXIN (V). (A-H**)** Cell lysates were subjected to western blot analysis using the indicated antibodies (left panels). The level of miR-125b-5p was detected by qRT-PCR (right panels). Error bars indicate mean ± SD of triplicate samples. **P<0.01; ***P<0.001. n.s indicates no significant difference.

mTORC1 is the most crucial downstream effector of TSC1/TSC2 complex 15. Since loss of TSC1/TSC2 complex induced upregulation of STAT3 as well as downregulation of miR-125b-5p, we speculated that mTORC1 participates in regulation of their expression. To determine this hypothesis, we first evaluated the effect of rapamycin, a specific mTORC1 inhibitor, on STAT3 and miR-125b-5p expression. As shown in the left panel of Figure 3A, rapamycin treatment in Tsc2-/- MEFs led to marked reduction in mTORC1 activity (p-S6) as well as STAT3 protein level. As expected, treatment with rapamycin significantly increased the expression of miR-125b-5p (Figure 3A right panel). Similarly, the expression of miR-125b-5p was dramatically upregulated while the expression of STAT3 was decreased in Tsc1-/- MEFs in response to rapamycin treatment (Figure 3B).

mTOR exists in two multi-protein complexes, rapamycin-sensitive mTORC1 and rapamycin-insensitive mTORC2 1. To further verify that it is indeed mTORC1 that mediates the positive regulation of STAT3 and the negative regulation of miR-125b-5p downstream of TSC1/TSC2 complex, we examined STAT3 and miR-125b-5p level in Raptor (a specific component of mTORC1) or Rictor (a specific component of mTORC2)-knockdown Tsc2-/- MEFs. As shown in Figure 3C and 3D, cells transfected with Raptor shRNAs exhibited decreased STAT3 and increased miR-125b-5p levels, while Rictor shRNAs had little effect on both the expression of STAT3 and miR-125b-5p. Furthermore, a similar result was obtained in Tsc1-/- MEFs with depletion of Raptor or Rictor (Figure 3E and 3F).

In addition to genetic loss of TSC1 or TSC2, mTORC1 activity can also be stimulated by the activation of PI3K/AKT signaling pathway, such as the inactivation of the tumor suppressor PTEN or the overexpression of the constitutively activated AKT 13. Not surprisingly, cells with either depleted PTEN or overexpression of constitutively activated AKT1 (myrAKT1) showed increased expression of STAT3 and decreased miR-125b-5p levels (Figure 3G and 3H). Taken together, hyperactivated mTORC1 signaling is responsible for the upregulation of STAT3 and downregulation of miR-125b-5p in Tsc1-/- or Tsc2-/- MEFs.

### mTORC1 upregulates STAT3 through downregulation of miR-125b-5p

**Figure 4:**
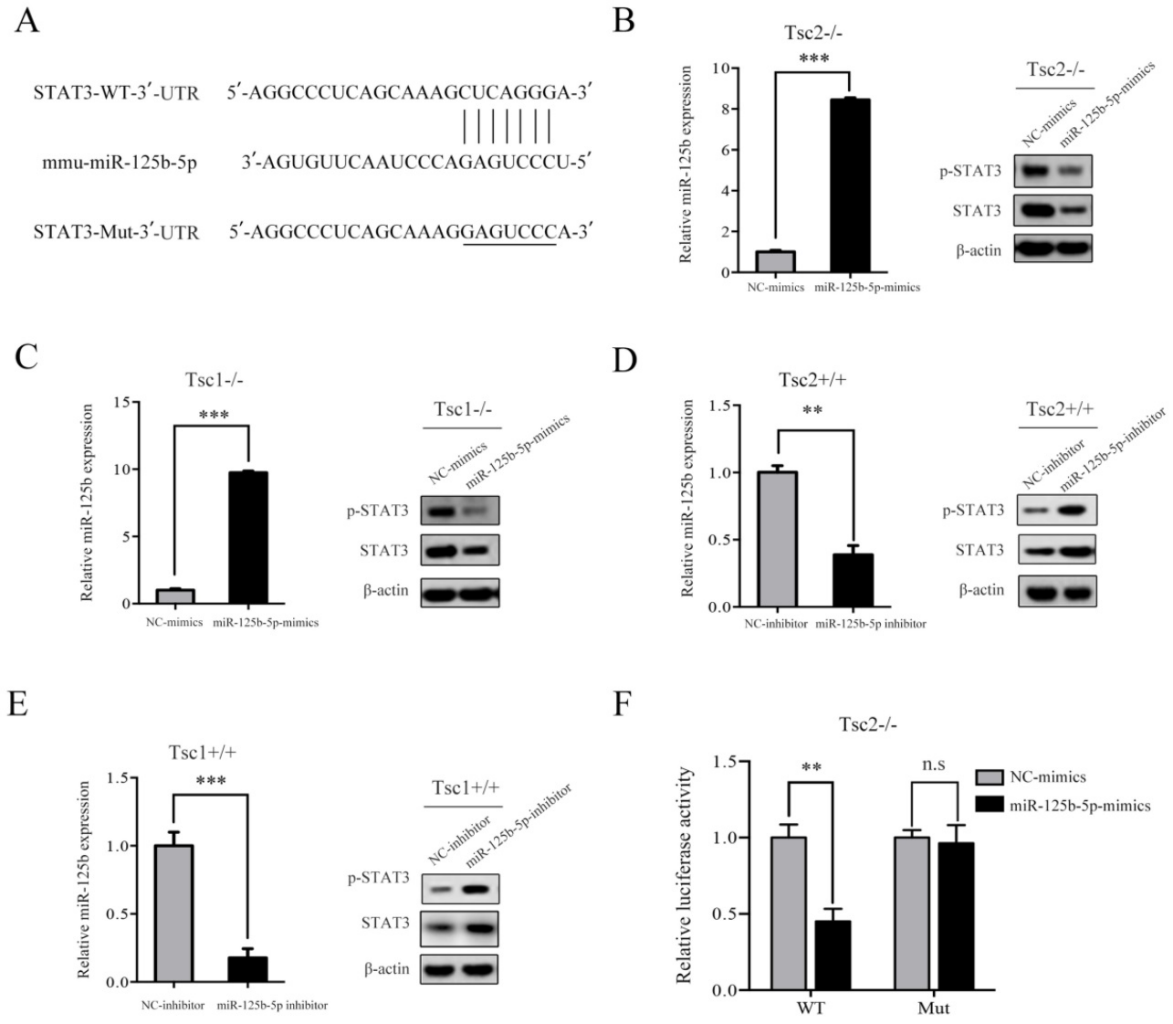
** mTORC1 upregulates STAT3 through inhibition of miR-125b-5p.** (A) Sequence alignment of predicted miR-125b-5p within the mouse STAT3 3´-UTR and its mutated sequence for luciferase reporter assay. (B, C) Tsc2-/- MEFs (B) and Tsc1-/- MEFs (C) were transfected with the miR-125b-5p mimics or negative control (NC-mimics). (D, E) Tsc2+/+ MEFs (D) and Tsc1+/+ MEFs (E) were transfected with the miR-125b-5p inhibitor or negative control (NC-inhibitor). (B-E) The expression level of miR-125b-5p of the indicated cells was analyzed by qRT-PCR (left panels). The expression of STAT3 in the indicated cells was examined by western blotting (right panels). (F) Luciferase reporter assay was performed in Tsc2-/- MEFs that were co-transfected with miR-125b-5p mimics or NC-mimics together with reporter vectors containing STAT3 3´-UTR or mutated STAT3 3´-UTR. Relative luciferase activities are presented. Data indicate mean ± SD of triplicate samples. **P<0.01; ***P<0.001. n.s indicates no significant difference.

Given that hyperactivated mTORC1 led to upregulated expression of STAT3 in parallel with downregulated expression of miR-125b-5p, it is likely that mTORC1 increases the expression of STAT3 through suppression of miR-125b-5p. To test this possibility, we searched for the putative target genes of miR-125b-5p using bioinformatics tools, such as TargetScan and miRanda. The analysis of the 3´-UTR of the STAT3 mRNA revealed a potential binding site of miR-125b-5p, which indicated that the existence of a regulative relationship between miR-125b-5p and STAT3 (Figure 4A). These data prompted us to examine whether miR-125b-5p can inhibit the expression of the endogenous STAT3 protein in cells with dysregulated mTORC1. As depicted in the left panel of Figure 4B, transfection of miR-125b-5p mimics into Tsc2-/- MEFs resulted in a substantial increase of miR-125b-5p expression compared to miR-NC-transfected cells. As expected, overexpression of miR-125b-5p markedly reduced the endogenous STAT3 protein levels in Tsc2-/- MEFs (Figure 4B right panel). A similar result was obtained in Tsc1-/- MEFs with overexpressed miR-125b-5p (Figure 4C). In contrast, transfection of miR-125b-5p inhibitor led to a significant decrease in miR-125b-5p expression as well as upregulated expression of STAT3 in Tsc2+/+ or Tsc1+/+ MEFs (Figure 4D and 4E). Therefore, mTORC1 upregulates STAT3 through inhibition of miR-125b-5p.

To further assess whether STAT3 is a direct target of miR-125b-5p, a luciferase activity assay was performed. A fragment of the 3´-UTR (STAT3-WT) and the mutant 3´-UTR (STAT3-Mut) were cloned into the GP-miRGLO firefly luciferase vector. Tsc2-/- MEFs were co-transfected with STAT3-WT or STAT3-Mut, miR-125b-5p mimics or NC, and pRL-TK luciferase reporters. As shown in Figure 4F, miR-125b-5p was able to markedly decrease the relative luciferase activity of STAT3-WT in Tsc2-/- MEFs, whereas that in the cells transfected with STAT3-Mut was not reduced. Collectively, STAT3 is a direct downstream target of miR-125b-5p.

### Overexpression of miR-125b-5p inhibits the proliferation and tumor growth of cells with hyperactivated mTORC1

**Figure 5:**
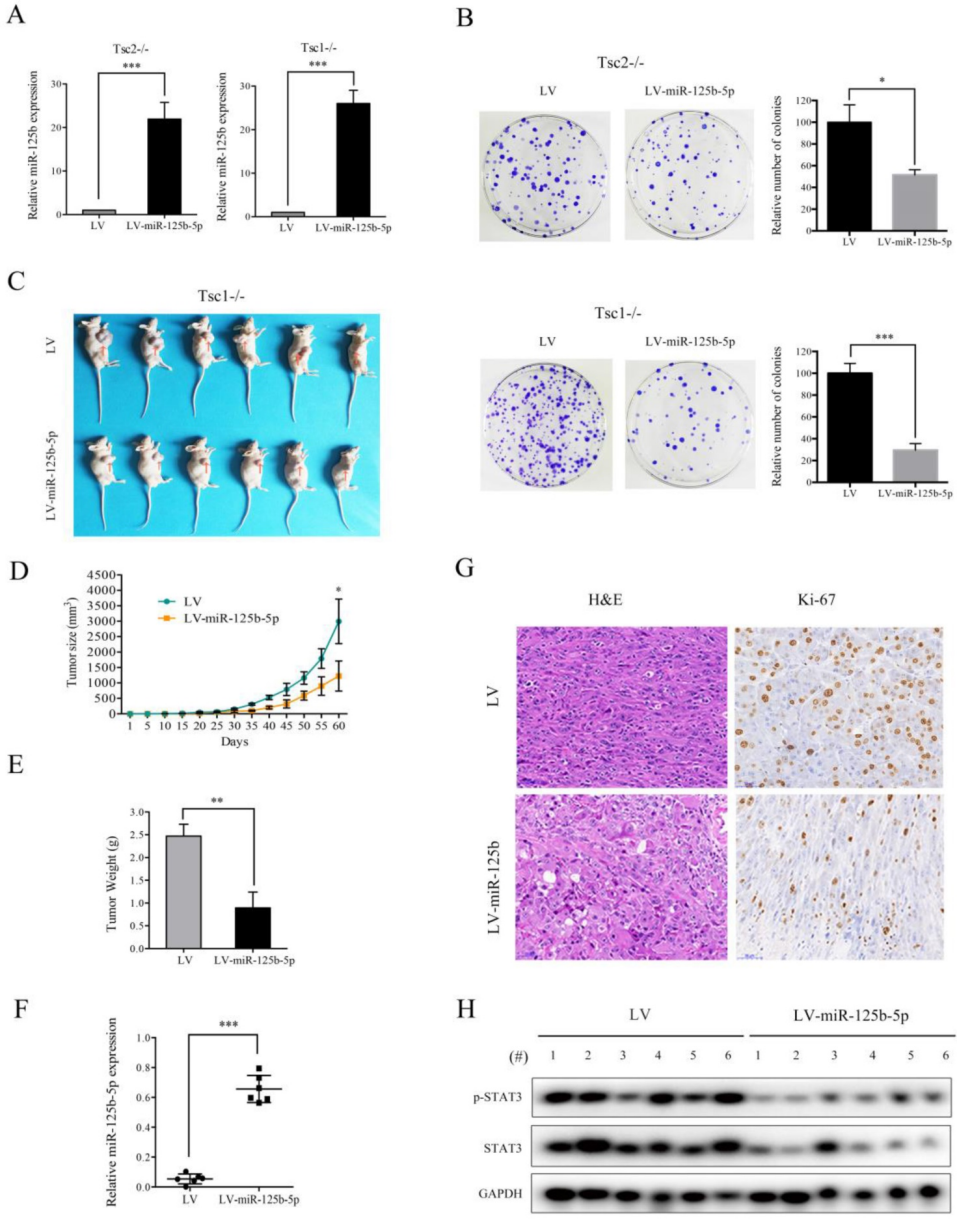
** miR-125-5p inhibits the colony formation, and *in vivo* tumorigenicity of Tsc2-/- or Tsc1-/- MEFs.** (A) Tsc2-/- or Tsc1-/- MEFs were infected with lentivirus harboring the GV369 vector encoding miR-125b-5p (LV-miR-125b-5p) or the empty vector (LV). The expression of miR-125-5p in these cells was determined by qRT-PCR. (B) The colonies formed by the indicated cells were stained and counted. Representative images (left panels) and quantifications (right panels). (C-H) Tsc1-/- MEFs transduced with LV-miR-125b-5p or LV lentiviruses were inoculated subcutaneously into nude mice, following by monitoring for tumor growth. (C) Tumor pictures. (D) Tumor volumes at different times. (E) Tumor weight. (F) miR-125-5p levels in the indicated tumor tissues were determined by qRT-PCR. (G) H&E and immunohistochemical staining of the indicated tumor tissues (×200). Representative images were presented. (H) Tumor tissues derived from Tsc1-/- MEFs transduced with LV-miR-125b-5p or LV lentiviruses were subjected to immunoblotting with the indicated antibodies. *P<0.05; **P<0.01; ***P<0.001.

mTORC1 signaling plays a crucial role in promoting cell proliferation and tumor growth 1. Since miR-125b-5p expression was significantly downregulated by mTORC1, we speculated that miR-125b-5p may exert suppressive effects on cell proliferation and tumorigenesis. Successful overexpression of miR-125b-5p in Tsc2-/- or Tsc1-/- MEFs was confirmed by qRT-PCR (Figure 5A). As expected, the ectopic expression of miR-125b-5p markedly suppressed the proliferation of Tsc2-/- or Tsc1-/- MEFs, as demonstrated by the colony formation assays (Figure 5B). To further investigate the role of miR-125b-5p on cell growth *in vivo,* Tsc1-/- MEFs transfected with lentiviral vector encoding miR-125b-5p or empty vector were subcutaneously injected into the right anterior armpit of nude mice, and then tumor growth was monitored. As depicted in Figure 5C-E, miR-125b-5p significantly inhibited the tumorigenesis of Tsc1-/- MEFs. The increased miR-125b-5p expression in the tumor samples derived from miR-125b-5p-overexpressing Tsc1-/- MEFs was confirmed by qRT-PCR (Figure 5F). Furthermore, IHC analysis demonstrated that the staining of proliferation maker Ki-67 was much weaker in tumor tissues derived from miR-125b-5p-overexpressing Tsc1-/- MEFs than those in the control counterpart (Figure 5G). Therefore, miR-125b-5p negatively regulates the cell proliferation and tumor growth of cells with aberrantly activated mTORC1 signaling.

### STAT3 is a functional target of miR-125b-5p downstream of mTORC1 signaling

**Figure 6:**
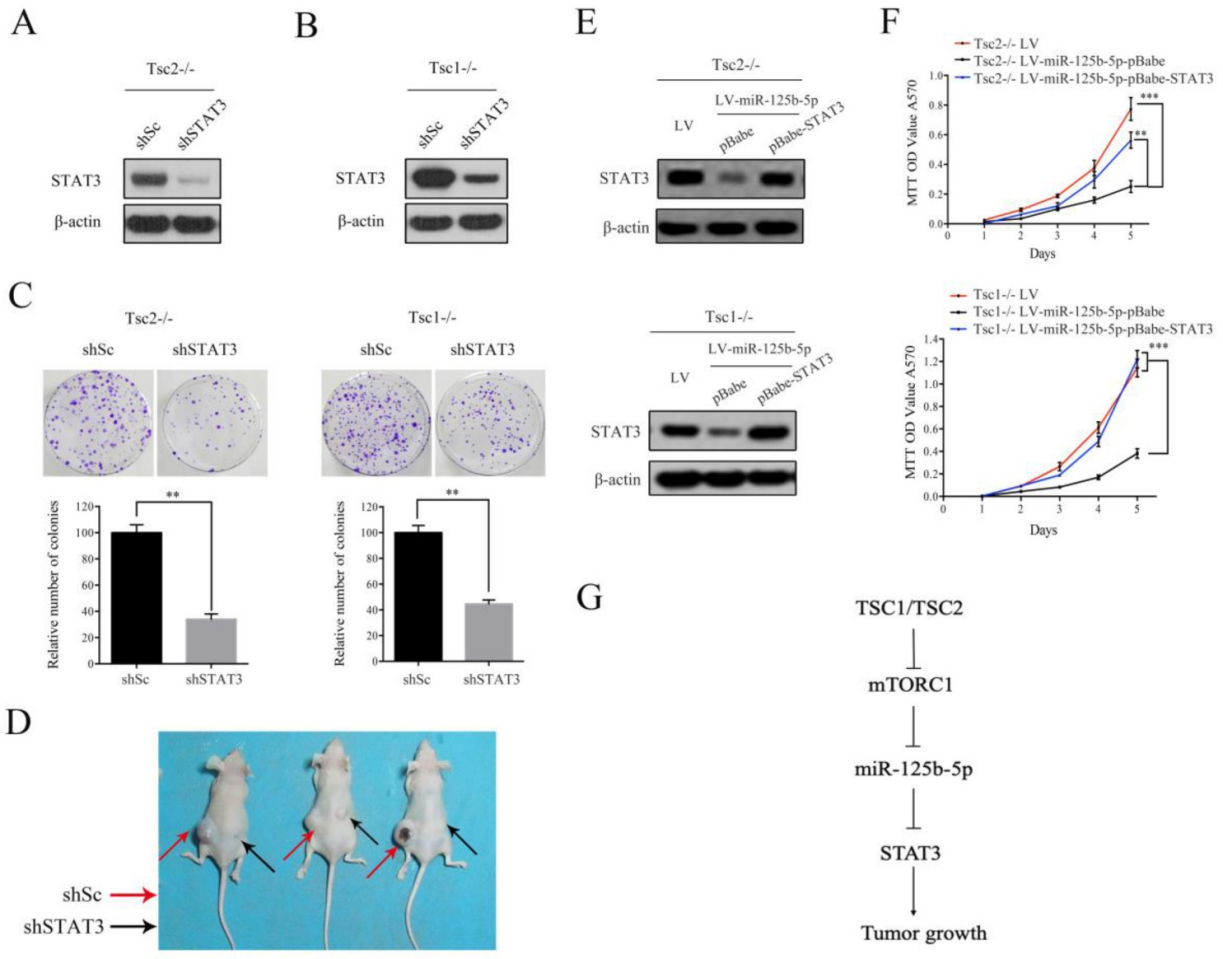
** STAT3 is a functional target of miR-125b-5p downstream of mTORC1 signaling.** (A, B) Tsc2-/- MEFs (A) and Tsc1-/- MEFs (B) were infected with lentivirus expressing shRNAs targeting STAT3 (shSTAT3) or a control shRNA (shSc). Cell lysates were subjected to immunoblotting with the indicated antibodies. (C) The colonies formed by the indicated cells were stained and counted. Representative images (upper panels) and quantifications (lower panels). (D) Tsc2-/- MEFs transduced with shSTAT3 or shSc lentiviruses were inoculated subcutaneously into nude mice, following by monitoring for tumor growth. 60 days after inoculation, mice were sacrificed and photographed. (E, F) LV-miR125b-5p expressing Tsc2-/- or Tsc1-/- MEFs were infected with pBabe or pBabe-STAT3 retroviruses. (E) Cell lysates of the indicated cells were subjected to western blot analysis. (F) The proliferation of the indicated cells was examined by MTT assay. (G) Schematic illustration of the TSC1/TSC2/mTORC1 signaling pathway promoted tumor growth through the miR-125b-5p/STAT3 network. Error bars indicate mean ± SD of triplicate samples. **P<0.01; ***P<0.001.

As shown in Figure 5H, decreased expression of STAT3 and a concomitant downregulation of p-STAT3 were observed in tumor tissues derived from miR-125b-5p-overexpressing Tsc1-/- MEFs compared to the corresponding control cells. We next investigated whether the anti-proliferative effect of miR-125b-5p was mediated by STAT3 in mTORC1-activated cells. A lentiviral vector expressing shRNA for STAT3 was transduced to Tsc2-/- MEFs and subsequent western blot analysis revealed that the shRNA for STAT3 dramatically suppressed expression of STAT3 (Figure 6A). Colony formation assays further showed that STAT3 depletion led to a decrease in cell proliferation (Figure 6C left panel). Similar results were obtained in Tsc1-/- MEFs expressing shSTAT3 (Figure 6B and 6C right panel). Furthermore, we injected Tsc2-/- MEFs expressing shSTAT3 or shSc subcutaneously into nude mice. As shown in Figure 6D, Tsc2-/- MEFs expressing shSTAT3 had reduced tumorigenic capacity in comparison with the control cells. Thus, depletion of STAT3 mimicked the effect of miR-125b-5p overexpression on Tsc2-/- or Tsc1-/- MEFs. To further establish the functional relationship between STAT3 and miR-125b-5p, we ectopically expressed STAT3 in miR-125b-5p-overexpressing Tsc2-/- MEFs (Figure 6E upper panel). As shown in the upper panel of Figure 6F, overexpression of STAT3 rescued the compromised cell proliferation driven by miR-125b-5p overexpression in Tsc2-/- MEFs. Consistent results were obtained in miR-125b-5p-overexpressing Tsc1-/- MEFs when STAT3 was overexpressed (Figure 6E lower panel and 6F lower panel). Taken together, these data revealed that miR-125b-5p reduces the growth capacity of Tsc1- or Tsc2-deficient cells at least partially through suppression of STAT3 expression.

## Discussion

mTORC1, a major regulator of cell growth, differentiation and cell metabolism, is often deregulated in malignancies 1. Notably, hyperactivated mTORC1, caused by loss-of-function mutations in the *TSC1* or *TSC2* gene, is considered to be the main reason of tuberous sclerosis complex (TSC), an autosomal dominant disease characterized by formation of benign tumors in multiple organs 6. Currently, the mTORC1 inhibitors, rapamycin as well as its analogues have been approved for the treatment of several types of human cancer, such as renal cell carcinoma, mantle cell lymphoma, and some TSC-related tumors, including lymphangioleiomyomatosis, angiomyolipomas, and subependymal giant cell astrocytomas 6, 16-18. Numerous studies indicate that mTORC1 signaling pathway is regulated by miRNAs 10. However, little is known about how miRNAs function in the dysregulated mTORC1-mediated tumorigenesis. Recently, Shen *et al.* showed that mTOR suppressed the expression of ataxia telangiectasia mutated (ATM) protein through upregulating miR-18a and miR-421 19. Subsequently, the dampened ATM led to genomic instability and tumorigenesis in the context of childhood sarcoma 19. Ji *et al.* reported that mTOR-mediated miR-212 upregulation led to suppression of autophagy in colorectal adenoma cells 20. Here we demonstrated that hyperactivated mTORC1 in Tsc2-/- or Tsc1-/- MEFs led to significantly downregulation of miR-125b-5p (Figure 3). Ectopic expression of miR-125b-5p inhibited the growth of cells with hyperactivated mTORC1 both *in vitro* and *in vivo* (Figure 5). Our finding indicates that miRNAs is involved in tumorigenesis driven by mTORC1, thus suggesting that miR-125b-5p is a potential therapeutic target of the cancers with hyperactivated mTORC1.

In recent years, emerging evidence indicate that miR-125b-5p is dysregulated in multiple types of cancer, including breast cancer, laryngeal squamous cell carcinoma, esophageal squamous cell carcinoma, and multiple myeloma 21-25. For example, Li reported that miR-125b-5p which was significantly downregulated in breast cancer cell lines, functioned as a tumor suppressor through targeting KIAA1522 21. Morelli *et al.* demonstrated that the expression of miR-125b-5p was decreased in multiple myeloma, and its ectopic expression impaired the growth and survival of multiple myeloma cells 25. Our study suggests that the decreased miR-125b-5p expression contributes to the accelerated cell proliferation and tumor growth driven by aberrantly activated mTORC1 signaling. Our findings reveal a novel molecular mechanism of mTORC1-activated carcinogenesis, and indicate that miR-125b-5p is a tumor suppressor. Given that mTORC1 is frequently activated in breast cancer and multiple myeloma 22, 26, it is possible that hyperactivated mTORC1 signaling inhibits the expression of miR-125b-5p and promotes cell growth and tumorigenesis in these types of cancer. However, the underlying mechanisms of miR-125b-5p regulation by mTORC1 remain unknown. It was reported that c-myc repressed miR-125b-5p transcription 27. Since c-myc is a well-identified downstream effector of mTORC1 28-30, it is possible that mTORC1 inhibits miR-125b-5p expression through upregulation of c-myc. Future works are required to explore this possibility.

Numerus studies indicate that STAT3 is implicated in tumor cell proliferation, invasion, and metastasis in human cancers 12. In response to upstream signals, such as cytokines and growth factors, STAT3 is phosphorylated at Tyr^705^, which facilitates its homo-dimerization and nuclear translocation to promote the transcription of downstream target genes whose promoters contain STAT3 binding sites 31. Additionally, STAT3 can be phosphorylated by numerous kinases on Ser^727^, a site that enhances the transcriptional activity of STAT3 31. STAT3 is a known downstream target of AKT/mTORC1 signaling pathway 5. mTOR was shown to phosphorylate STAT3 at Ser^727^, and then promote its transcriptional activity in neuroblastoma cells 32. Our previously study suggested that mTORC1 activated STAT3 by inducing the phosphorylation of STAT3 at Tyr^705^, leading to the inhibition of cell differentiation and upregulation of glycolysis 4, 13. However, the regulation of STAT3 expression remains less characterized. In this study, we found that activated mTORC1 can also increase the expression of STAT3 in addition to inducing STAT3 phosphorylation (Figure 3). Interestingly, the mRNA level of STAT3 remained stable in response to mTORC1 activation. We demonstrated that mTORC1 upregulated STAT3 expression through the suppression of miR-125b-5p (Figure 4). Moreover, knockdown of STAT3 diminished the proliferation of the cells with hyperactivated mTORC1 both *in vitro* and *in vivo* (Figure 6). Thus, mTORC1 modulates STAT3 at multiple levels and STAT3 is an ideal therapeutic target of mTORC1-mediated tumors.

In summary, we demonstrated that hyperactive mTORC1 signaling contributes to accelerated cell proliferation and tumor growth through the suppression of miR-125-5p expression. Furthermore, miR-125b-5p exerts an anti-proliferative effect in mTORC1-activated cells through STAT3 inhibition (Figure 6G). Our findings provide a novel molecular mechanism of the tumorigenesis driven by dysregulated mTORC1 signaling, suggesting miR-125b-5p is a therapeutic tool for treatment of mTORC1-related tumor.

## Figures and Tables

**Table 1:** The differentially expressed miRNAs between Tsc2-/- and Tsc2+/+ MEFs (Fold Change>2).

miRNAs	Fold Change Tsc2-/- vs Tsc2+/+	Expression
mmu-miR-146a	13.83	Up
mmu-miR-10a-5p	6.87	Up
mmu-miR-10b	6.36	Up
mmu-miR-130b	5.62	Up
mmu-miR-99a	3.71	Up
mmu-miR-1894-3p	3.56	Up
mmu-miR-503-5p	3.29	Up
mmu-miR-322-3p	3.29	Up
mmu-miR-322-5p	3.2	Up
mmu-miR-20a	2.28	Up
mmu-miR-17	2.16	Up
mmu-miR-720	2.1	Up
mmu-miR-351-5p	2.04	Up
mmu-miR-127	-2.1	Down
mmu-miR-134	-2.23	Down
mmu-miR-148a	-2.35	Down
mmu-miR-199a-5p	-2.36	Down
mmu-miR-132	-2.36	Down
mmu-miR-27a	-2.45	Down
mmu-miR-574-5p	-2.48	Down
mmu-miR-30b	-2.51	Down
mmu-miR-15a	-2.95	Down
mmu-miR-98	-2.68	Down
mmu-miR-541	-2.71	Down
mmu-miR-29a	-2.73	Down
mmu-miR-26b	-2.77	Down
mmu-miR-199a-3p	-2.77	Down
mmu-miR- let-7d*	-2.81	Down
mmu-miR-689	-2.85	Down
mmu-miR-143	-2.87	Down
mmu-miR-152	-2.97	Down
mmu-miR-382	-3.12	Down
mmu-miR-224	-3.18	Down
mmu-miR-667	-3.25	Down
mmu-miR-342-3p	-3.46	Down
**mmu-miR-125b-5p**	**-3.78**	**Down**
mmu-miR-199b-5p	-3.92	Down
mmu-miR-455	-4.32	Down
mmu-miR-495	-4.86	Down
mmu-miR-411*	-5.28	Down
mmu-miR-290-5p	-5.66	Down
mmu-miR-146b	-5.7	Down
mmu-miR-181d	-5.74	Down
mmu-miR-433	-6.11	Down
mmu-miR-434-3p	-6.73	Down
mmu-miR-206	-6.77	Down
mmu-miR-181b	-7.01	Down
mmu-miR-365	-7.94	Down
mmu-miR-1907	-9.13	Down
mmu-miR-376c-3p	-13.18	Down
mmu-miR-218	-26.91	Down
